# Infective myositis, an uncommon presentation of melioidosis: a case report and review of the literature

**DOI:** 10.1186/s13256-019-2321-3

**Published:** 2019-12-31

**Authors:** Nuwan Jayawardena, Udaya Ralapanawa, Prabhashini Kumarihamy, Thilak Jayalath, Shashi Prabhani Abeygunawardana, Nadisha Dissanayake, Priyantha Dissanayake, Jeevani Udupihille, Neelakanthi Ratnatunga, Chamara Dalugama

**Affiliations:** 1 Teaching Hospital Peradeniya, Peradeniya, Sri Lanka; 2 Department of Medicine, University of Peradeniya, Peradeniya, Sri Lanka; 3 Department of Radiology, University of Peradeniya, Peradeniya, Sri Lanka; 4 Department of Pathology, University of Peradeniya, Peradeniya, Sri Lanka

**Keywords:** Melioidosis, Atypical, Infective myositis, Treatment, Sri Lanka

## Abstract

**Background:**

Melioidosis is considered endemic in certain areas of the world. Musculoskeletal and soft tissue involvement are relatively uncommon presentations in melioidosis. We present a case of infective myositis in a patient with melioidosis in Sri Lanka, which is not considered an endemic country. Even though multiple cases of melioidosis have been reported with an increasing number in Sri Lanka, infective myositis secondary to melioidosis was not reported previously.

**Case presentation:**

A 60-year-old Sinhalese man with diabetes presented with fever of 4 months’ duration and a limp with a painful lump on the right side of the upper thigh of 2 months’ duration. He had been treated in a local hospital for community-acquired pneumonia 3 weeks prior to this admission, for which he had received intravenous meropenem and teicoplanin with intensive care unit admission. He had a 0.5-cm × 0.5-cm tender lump over the right vastus lateralis muscle, and contrast-enhanced computed tomography of the area showed an ill-defined, heterogeneously enhancing, hypodense area involving the vastus lateralis, vastus intermedius, and quadratus femoris, suggestive of infective myositis but without abscess formation. Histopathology of the muscle biopsied from the vastus lateralis showed suppurative inflammation of subcutaneous fat with connective tissue necrosis and muscle infiltrated by lymphocytes. These features are suggestive of infective myositis possibly due to melioidosis. Although the result of a culture taken from the muscle biopsy was negative, the patient’s antibody titer was strongly positive for melioidosis. He did not show any other areas with infected foci. He was treated with intravenous meropenem for 2 weeks and responded well. He was discharged with trimethoprim-sulfamethoxazole for 6 months as a maintenance therapy.

**Conclusion:**

Melioidosis is commonly an undiagnosed disease that has a wide variety of clinical presentations. Myositis in melioidosis is uncommon, and careful evaluation is mandatory to avoid misdiagnosis of this treatable but fatal disease. The clinician should have a high index of clinical suspicion, and further clinical and epidemiological studies are needed to determine the true burden of the disease.

## Background

Melioidosis, caused by the gram-negative bacillus *Burkholderia pseudomallei*, has a high case fatality [1]. Although Sri Lanka is not considered endemic, multiple cases have been reported with increasing numbers [2].

The clinician should have a high index of suspicion because the clinical presentation of melioidosis is not distinctive. Patients can present with acute septicemia to a chronic suppurative disease and/or with localized or disseminated infection [3]. Pneumonia is the most common presentation in melioidosis worldwide, and risk is increased in patients with diabetes mellitus, chronic obstructive airway disease, congestive cardiac failure, smoking, and alcoholism [4]. Hematogenous spread of the organism may cause skin and soft tissue infection [5]. Other internal organ involvement, such as liver and spleen, with abscess formation is common. Prostate involvement with abscess formation is also common in male patients [6, 7], and encephalomyelitis is another complication with significant morbidity and mortality [8]. Although uncommon, striated muscle inflammation is another manifestation of this condition [5].

Striated muscle inflammation or myositis in melioidosis is uncommon and can mimic other infections both clinically and radiologically [9]. Musculoskeletal melioidosis can be diagnosed only on the basis of immunological and bacteriological test results [9]. We report a case of infective myositis in a patient with melioidosis; this complication of melioidosis was not reported previously in the Sri Lankan literature.

## Case presentation

We report a case of a 60-year-old Sinhalese man from a rural area of central Sri Lanka who presented to a teaching hospital with on-and-off fever of 4 months’ duration and pain in the lateral aspect of the right upper thigh of 2 months’ duration. He had been admitted to a local hospital 3 weeks prior to admission to our hospital with a history of nearly 3 months of on-and-off fever and a painful lump at the lateral aspect of the thigh. He had been treated for multilobar pneumonia at the local health facility with intravenous meropenem and teicoplanin for 14 days, and he required intensive care unit management during the course of his illness. Although his pain and the size of the lump at the lateral aspect of the thigh decreased with treatment, the lump and pain over the area persisted even after he was discharged from the local hospital. Further, he experienced on-and-off fever again, though he was afebrile upon discharge from the local hospital. With time, he experienced difficulty in walking with increasing severity of pain in the lateral aspect of the right upper thigh, for which he sought medical advice. On admission to our hospital, he had high-grade fever, and he denied any respiratory or urinary symptoms. His bowel motions were normal with normal color and contour. He was a farmer and had a history of exposure to surface water. He was a nonsmoker and never used alcohol. He was diagnosed for the past 8 years with type 2 diabetes mellitus, which was treated with oral hypoglycemic drugs. He was not receiving any medication other than oral hypoglycemic drugs, and he denied recent use of steroids. He did not have any high-risk behaviors.

On examination, he was febrile (102 °F) and mildly pale but not icteric. He did not have enlarged peripheral lymph nodes or skin rashes. He had a small induration over the lateral aspect of his right upper thigh, which was tender. On further examination, lumpiness was detected deep in the indurated area, measuring 0.5 cm × 0.5 cm, which was firm and tender. Further, there was surrounding muscle tenderness. He did not have evidence of thrombophlebitis. He was hemodynamically stable, and the results of his respiratory and cardiovascular examinations were perfectly normal. His abdomen was soft, and he did not have hepatosplenomegaly. The result of his neurological examination, including bilateral fundi, was normal.

**Fig. 1:**
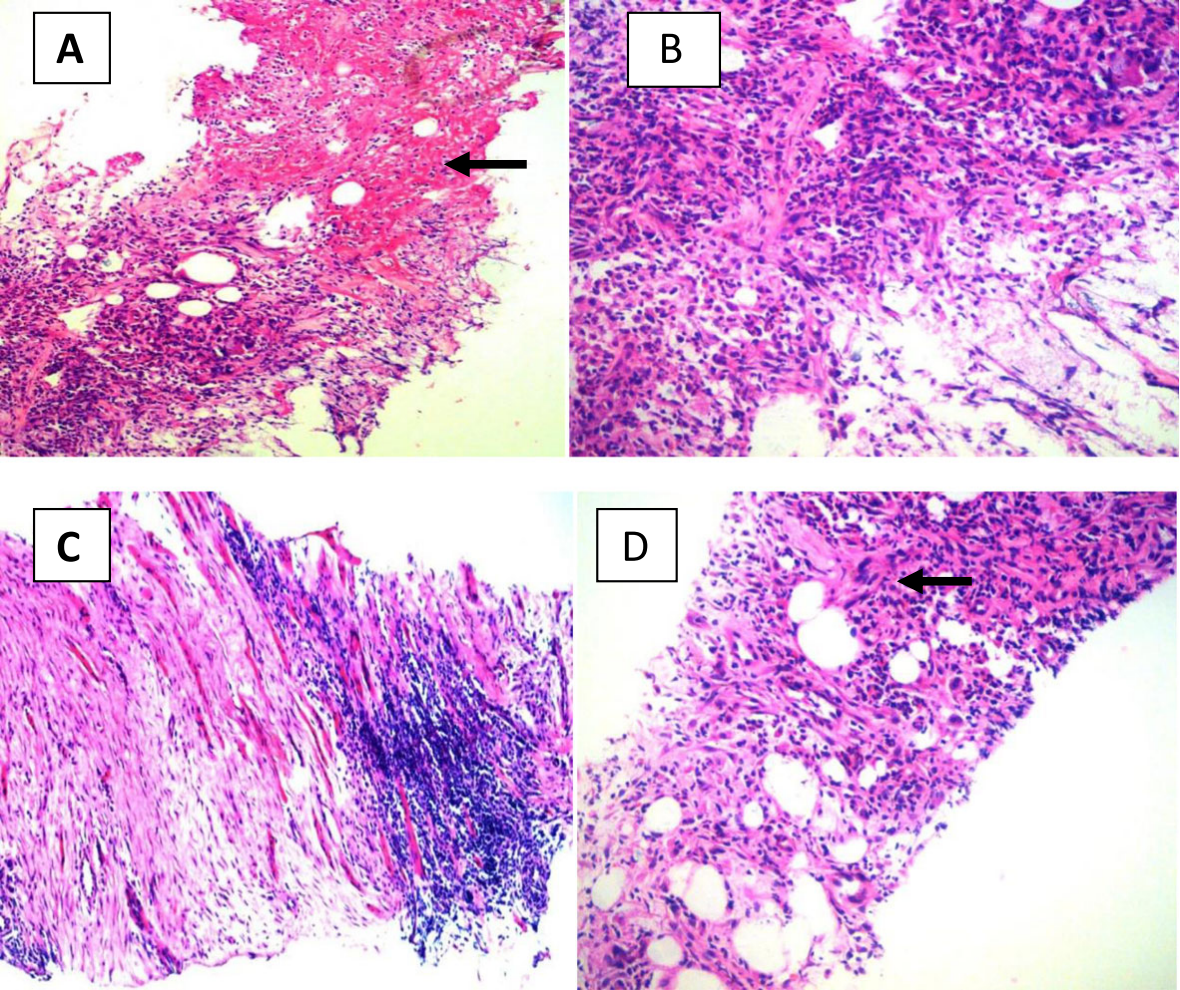
Hematoxylin and eosin (H&E)-stained biopsy of the affected muscle. **a** Coagulative necrosis in the muscle in subcutaneous tissue plane (*arrow*). Original magnification, × 100. **b** Tissue infiltrated with neutrophils, lymphocytes, and plasma cells. Original magnification, × 200. **c** Skeletal muscle tissue infiltrated with lymphocytes and areas of fibrosis. Original magnification, × 100. **d** A granuloma with Langhans-type giant cell in the subcutaneous tissue (*arrow*). Original magnification, × 200

His initial full blood count showed a white blood cell count of 15,500/mm^3^ with 80% neutrophils. His blood picture showed normochromic*,* normocytic anemia (hemoglobin 8.0 g/dl) with neutrophil leukocytosis, suggesting anemia of chronic disease but without any evidence of bone marrow infiltration. His initial C-reactive protein (CRP) level and erythrocyte sedimentation rate (ESR) were 170 mg/L and 70 mm in the first hour, respectively, and he had an elevated serum creatine kinase level. His liver enzymes were marginally elevated with alanine transaminase of 111 U/L and aspartate transaminase of 87 U/L, but his liver and renal function were normal, and his hemoglobin A1c was 7%. His antibodies for human immunodeficiency virus types 1 and 2 were negative. The findings of his chest x-ray, 2D echocardiogram, and ultrasound scan of the abdomen were normal. He was started on intravenous flucloxacillin and meropenem after blood cultures and serum were taken for melioidosis antibodies. Ultrasound scan of the right lateral thigh showed a lesion that extended up to the right-side femur, but an x-ray of the right femur was normal. Contrast-enhanced computed tomography of the right thigh showed an ill-defined, heterogeneously enhancing, hypodense area in the vastus lateralis, vastus intermedius, and quadratus femoris, suggestive of infective myositis but without abscess formation. The findings of contrast-enhanced computed tomography of the chest and abdomen were normal. The patient underwent ultrasound-guided muscle biopsy, which showed coagulative necrosis in the muscle/subcutaneous tissue; infiltration with neutrophils, lymphocytes, and plasma cells in the adjacent tissue; focal suppuration; granulomata with Langhans-type giant cells; and focal fibrosis in the muscle (Fig. 1). The histopathological features were suggestive of melioidosis. A muscle biopsy specimen was sent for bacterial culture and antibiotic sensitivity testing as well as culture for tuberculosis and melioidosis, and all results were negative. The results of an indirect hemagglutination assay for melioidosis antibodies were highly positive with a titer > 10,240. The patient received intravenous meropenem for 2 weeks and was started on eradication therapy with oral cotrimoxazole 960 mg 12-hourly after the intensive phase and continued for 6 months. He was clinically improving with reduced pain over the lateral aspect of the right thigh and was fever-free by day 7 of treatment. He was discharged after 14 days of treatment with meropenem. On discharge, the patient’s ESR was 40 mm in the first hour, and his CRP level was 15 mg/L. At his follow-up appointment after 2 weeks, he reported resolution of symptoms.

## Discussion

Melioidosis, caused by the gram-negative bacillus *B. pseudomallei*, is regarded as endemic to Southeast Asia and northern Australia and has a high case fatality rate [1]. Although Sri Lanka is not considered an endemic area, an increasing number of cases have been reported from various parts of the country [2]. The bacteria are found in contaminated water and soil and spread to humans and animals through direct contact with contaminated water.

Pneumonia is the most common presentation of melioidosis worldwide and is involved in nearly half of all cases. One study involving 624 patients with culture-positive melioidosis showed that 319 (51%) patients presented with pneumonia as the primary diagnosis [4]. The same study described congestive cardiac failure, chronic obstructive pulmonary disease, smoking, and diabetes mellitus as risk factors for presentation with primary pneumonia. Our patient had been treated for pneumonia requiring intensive care unit care 3 weeks prior to admission at our institution. This could be the primary presentation of melioidosis. Skin and soft tissue infections are a common manifestation of melioidosis. The presentation may be rapidly progressive, similar to necrotizing fasciitis [8]. Hematogenous spread is thought to be the main source of infection in the skin and soft tissue. Hematogenous spread of the bacteria to skin and subcutaneous tissue would have caused the infective myositis in our patient as well. However, progression to abscess formation and necrotizing fasciitis would have been halted by initial treatment with meropenem. Striated muscle inflammation or myositis in melioidosis is uncommon. Musculoskeletal melioidosis mimics other infections both clinically and radiologically; importantly, diagnosis can be established only by bacteriological and immunological studies [9]. If affected, surgical drainage is often required together with a long course of intravenous antibiotics [1].

Involvement of other internal organs, especially liver and spleen, with abscess formation is common. Our patient had elevated liver enzymes, but ultrasound or computed tomography of the abdomen did not show any evidence of liver or splenic abscesses. Prostatic abscess is common in Australia, involving 18% of male patients, and drainage is commonly required in these patients [6, 7]. Our patient did not have features of prostatism. Encephalomyelitis is another uncommon complication of the disease, but with significant morbidity and mortality [8].

Diagnosis of melioidosis can be challenging. It can mimic other infections, and clinical suspicion is necessary in a patient presenting with atypical symptoms. Isolation of *B. pseudomallei* from body fluids of the patient remains the gold standard in diagnosis [1]. The blood culture and muscle biopsy culture results for melioidosis were negative in our patient. He was treated with intravenous meropenem for 2 weeks at the local hospital and 4 days in our hospital prior to muscle biopsy, which would have led to the negative culture results. Other tests that help in diagnosis are serological with antigen or antibody detection. Antibody detection is the method used in our patient. Indirect hemagglutination assay is used for antibody detection, but some studies have shown low sensitivity and specificity, especially in endemic areas [10].

In our patient’s case, the history of severe multilobar pneumonia responding to meropenem in a diabetic farmer and strongly positive serology in a nonendemic country led us to the diagnosis of melioidosis. He was treated with intravenous meropenem according to guidelines [2, 11] for the management of the intensive phase. Intravenous ceftazidime (2 g 6-hourly) [12] and meropenem (1 g 8-hourly) are agents of choice in the intensive phase. A study involving 217 patients with melioidosis showed that the use of meropenem was associated with outcomes at least as good as those obtained with ceftazidime [13]. Although our patient was treated in a local hospital with meropenem for pneumonia, melioidosis was not suspected at that time. Hence, he was not given the eradication treatment, and this may be the reason for his persisting disease. He responded to meropenem and was discharged with trimethoprim-sulfamethoxazole according to recommendations.

Even though we could not confirm the diagnosis of melioidosis with a positive culture or other standard method, circumstantial evidence was very strong for melioidosis in this case. Negative blood culture and muscle biopsy culture were expected because he had already been treated with relevant antibiotics at the time of sampling. The clinical presentation, complete resolution of symptoms, and normalization of laboratory results, including creatine kinase, after treatment of melioidosis are strong supporting evidence for the diagnosis of myositis due to melioidosis.

The clinician should have a high index of clinical suspicion because the clinical presentation of melioidosis is not distinctive. Improved and speedier laboratory diagnosis is needed to ensure early administration of appropriate therapy and to improve prognosis.

## Conclusion

Melioidosis is a commonly undiagnosed disease that has a wide variety of clinical presentations. Published case reports are likely to represent only the “tip of the iceberg” of this fatal but treatable infection. It is likely that melioidosis is more prevalent in Sri Lanka than currently perceived. The clinician should have a high index of clinical suspicion, and further clinical and epidemiological studies are needed to determine the true burden of the disease.

## Data Availability

Not applicable.

## Abbreviations

ALT: Alanine transaminase
AST: Aspartate transaminase
CECT: Contrast-enhanced computed topography
CRP: C-reactive protein
ESR: Erythrocyte sedimentation rate
FBC: Full blood count
ICU: Intensive care unit
